# Supplementary material for: Bladder cancer variants share aggressive features including a CA125+ cell state and targetable TM4SF1 expression
Source: Nat Commun. 2025 Jun 17;16:5312. doi: 10.1038/s41467-025-59888-8 (PMC12174346; doi:10.1038/s41467-025-59888-8)

Bladder cancer variants share aggressive features including a CA125+ cell state and targetable TM4SF1 expression

Supplementary Tables and Figures

Supplementary Table 1. Clinical and pathologic characteristics of patients and sequenced bladder cancer tissues. All self-reported patient demographics (age and biological sex) were collected at time of surgery. Consent was obtained for individual-level data sharing. TURBT = Transurethral resection of bladder tumor, HV = histologic variant, CIS = carcinoma in situ.

ID	Age Range	Sex	Neoadjuvant therapy	Operation	Stage	HV subtype 1	HV subtype 2	CIS present
UC01	60-70	F	chemotherapy	cystectomy	T2			no
UC02	70-80	M	immunotherapy	cystectomy	Ta			no
UC03	70-80	M	none	TURBT	T1			no
UC04	70-80	F	none	cystectomy	T1			yes
VAR01	60-70	M	none	cystectomy	T2	micropapillary		yes
VAR02	80-90	M	chemotherapy	TURBT	T2	micropapillary		no
VAR03	50-60	M	none	TURBT	T1	pleomorphic giant cell-like	micropapillary	no
VAR04	80-90	M	none	TURBT	T2	nested		no
VAR05	60-70	M	chemotherapy	cystectomy	T4	nested		yes
VAR06	80-90	M	none	cystectomy	T2	lymphoepithelioma-like		yes
VAR07	40-50	M	chemotherapy	cystectomy	T2	squamous differentiation		yes
VAR08	60-70	M	none	cystectomy	T4	plasmacytoid		no
VAR09	80-90	F	chemotherapy	cystectomy	T3	small cell	micropapillary	no
VAR10	60-70	F	chemotherapy	cystectomy	T4	pure squamous		no
VAR11	60-70	M	chemotherapy	cystectomy	T4	squamous differentiation	sarcomatoid differentiation	no

Supplementary Figure 1. Representative H&E stains from each sequenced tumor. Scale bars = 50 μm.

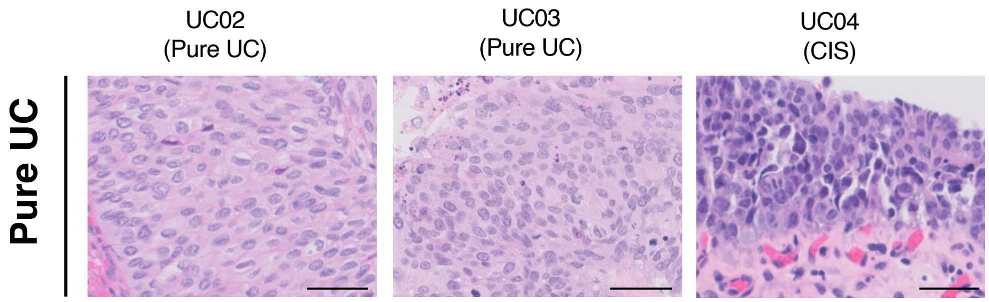

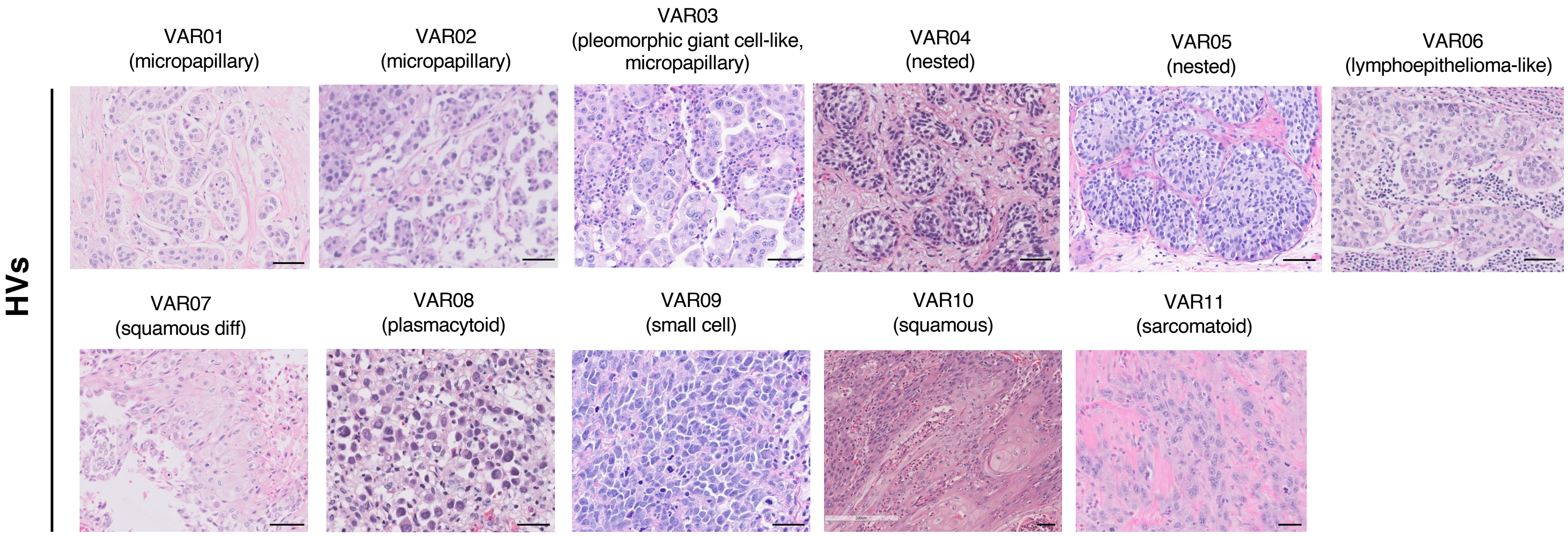

Supplementary Figure 2. Single cell dataset of variant and pure urothelial tumor epithelial cells. (A) Tissue acquisition and scRNA-seq workflow for primary bladder tumors. (B) UMAP of full dataset color-coded by broad cell type. (C) Bar chart of cell counts obtained from each patient/tumor.

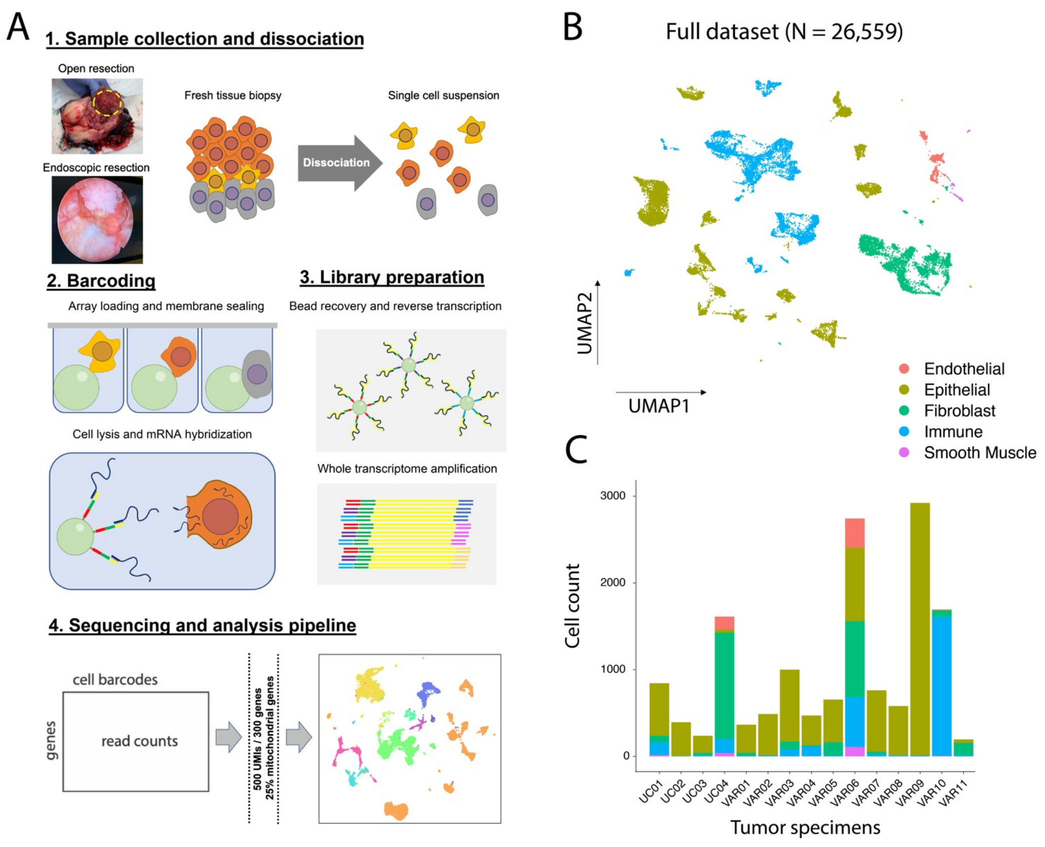

Supplementary Figure 3. Annotation of cell types and confirmation of tumor content. (A) Feature plots of genes used to determine top-level annotations for epithelial cells (EPCAM, KRT7), immune cells (PTPRC), fibroblasts (DCN), smooth muscle (ACTA2), and endothelial cells (SELE). (B) InferCNV analysis of all tumor epithelial cells using tumor microenvironment components for comparison.

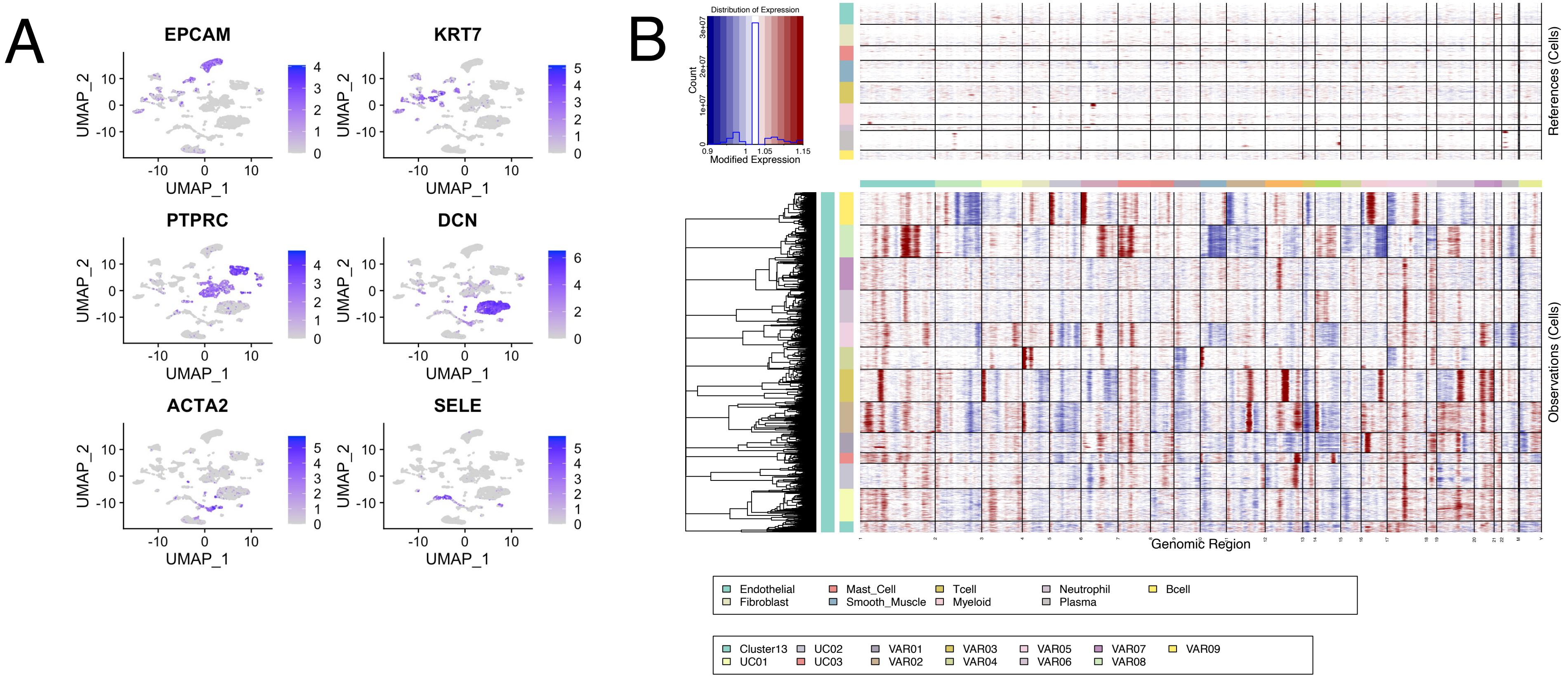

Supplementary Figure 4. Enrichment of non-urothelial genes and pathways in HVs. A. Key genes in plasma cell development. B. Plasma cell gene enrichment in tumor cells by tumor. C. Expression of small cell lung cancer subtype genes in VAR09. D. Small cell lung cancer gene enrichment in tumor cells by tumor.

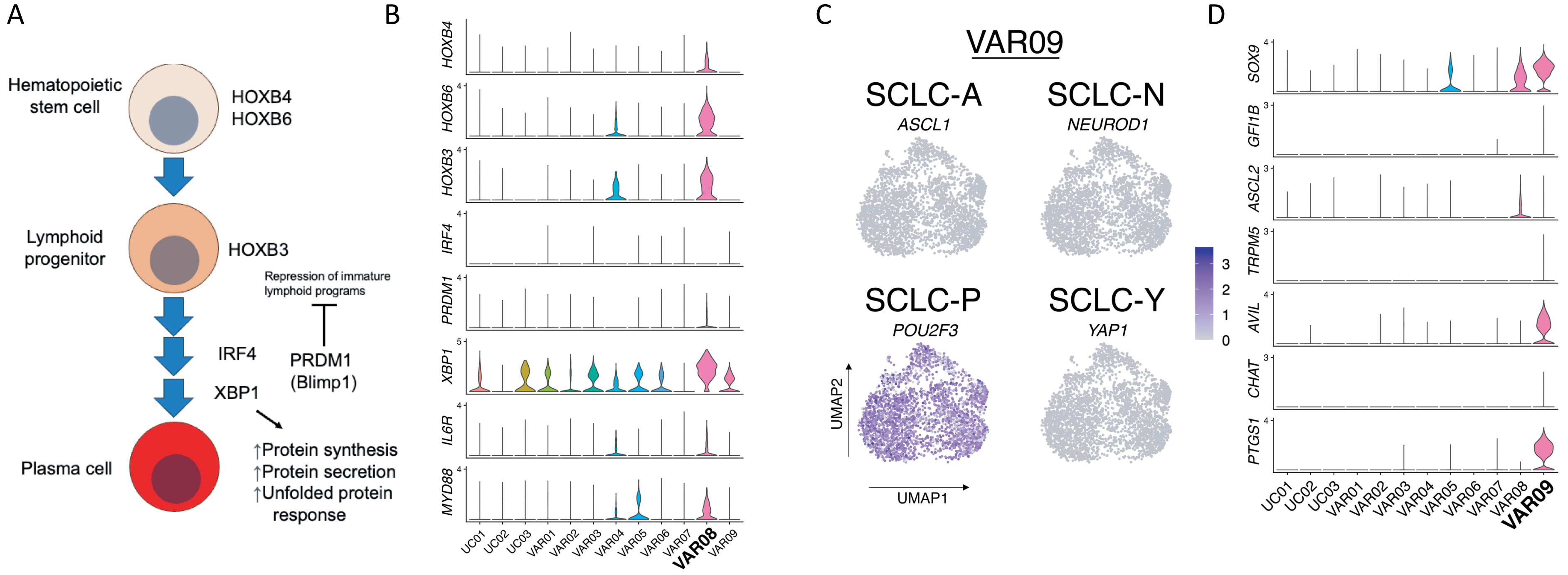

Supplementary Figure 5. Location of Cluster 13 cells on integrated UMAP of full dataset. Cluster 13 cells, which were identified via subset analysis of tumor epithelial cells in a non-integrated dataset, are highlighted in red.

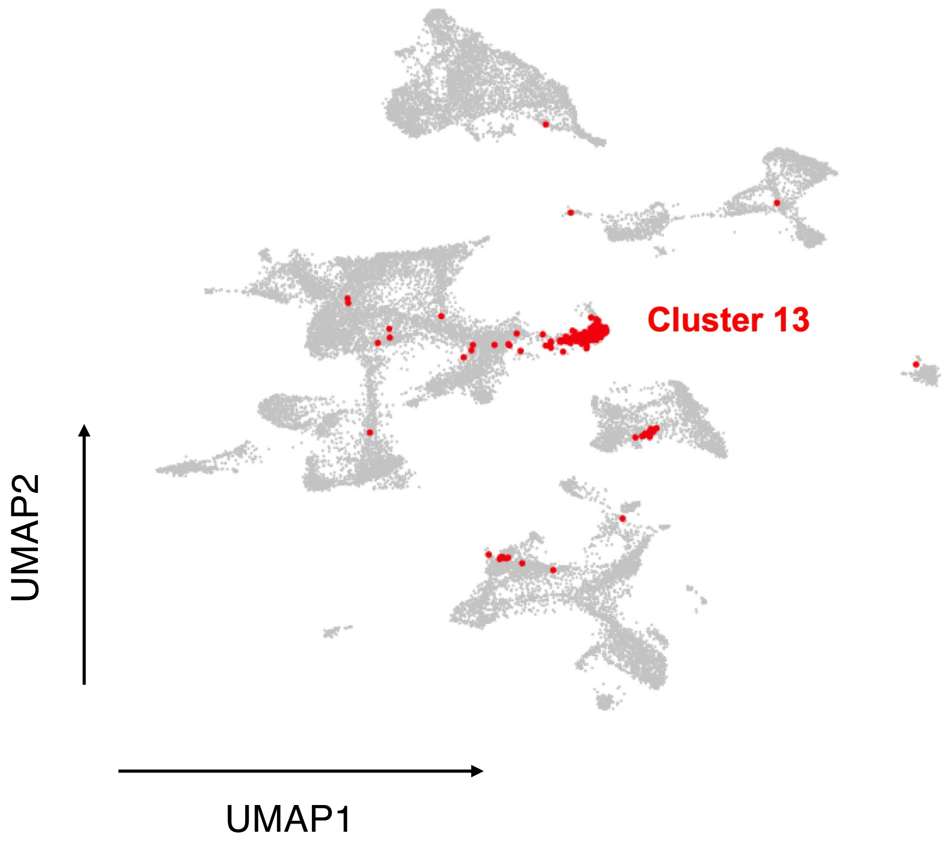

Supplementary Figure 6. Negative CA125 (MUC16) staining in variant and pure UC tumor components. Scale bar = 50 μm. UC = Urothelial carcinoma. CIS = Carcinoma in situ. H&E = Hemotoxylin and eosin. IHC = Immunohistochemistry.

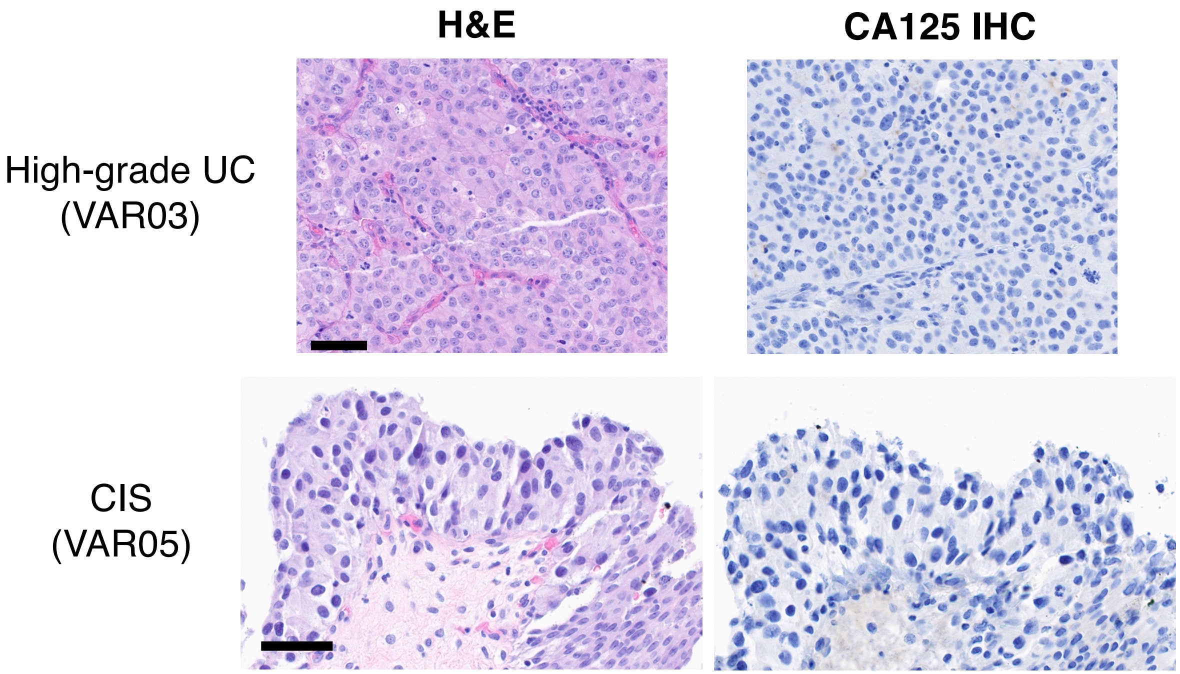

Supplementary Figure 7. Detection of Cluster 13 signature in an external scRNAseq data set (Chen et al). (A) Feature plot of Cluster 13 signature gene set enrichment in tumor epithelial cells (*N* = 6,271 cells). (B) Feature plots demonstrating expression of individual Cluster 13-defining genes.

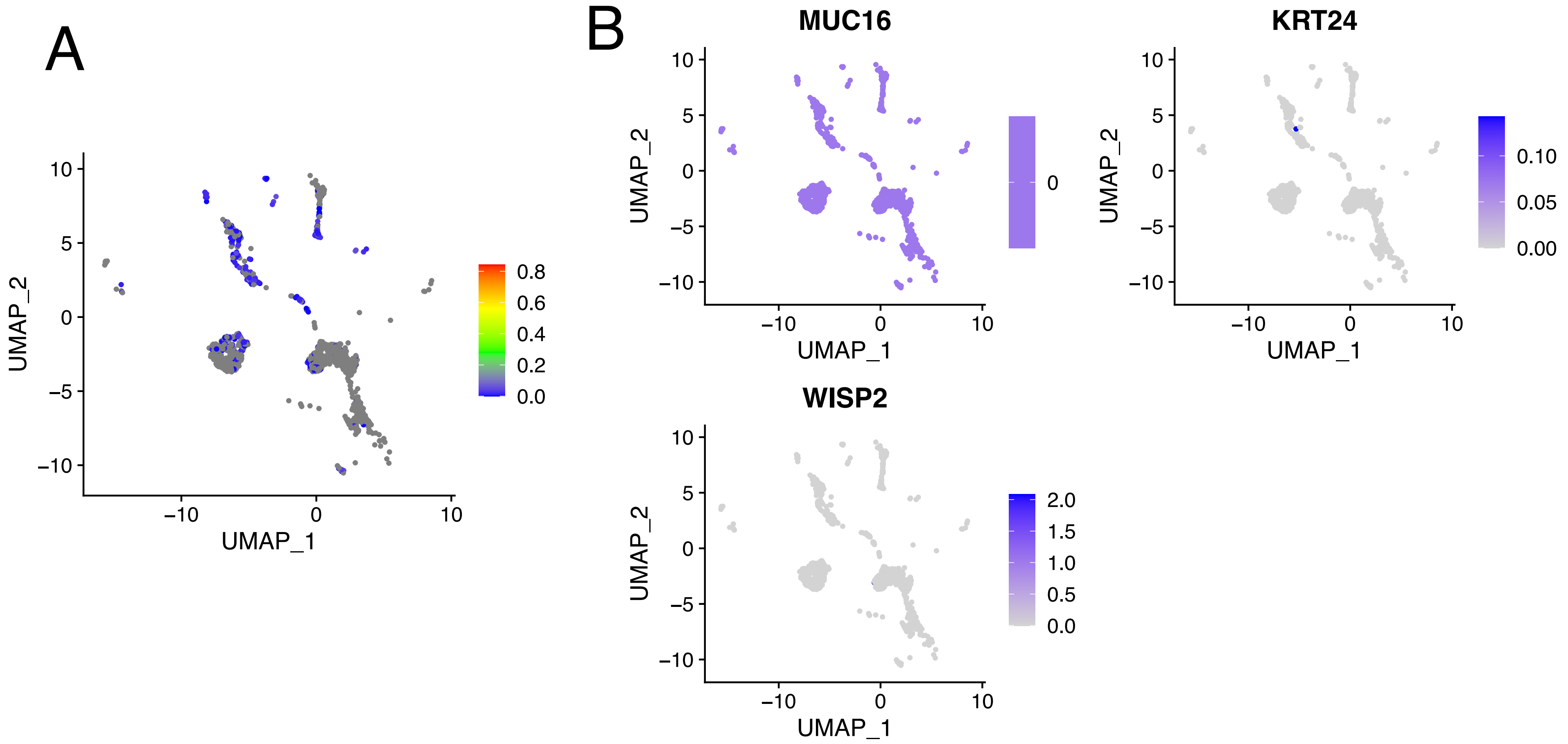

Supplementary Figure 8. Enrichment of bladder cancer stem cell signature (*PROM1* (CD133), *POU5F1* (Oct4), *SOX2*, *ALDH1A1*, *SOX4*, *EZH2*, *YAP1*, *CD44*, and *KRT14*) along the pseudotime for VAR01, VAR03, VAR05, VAR06, and VAR07. Cluster 13 cells were arbitrarily chosen as starting point of pseudotime trajectory.

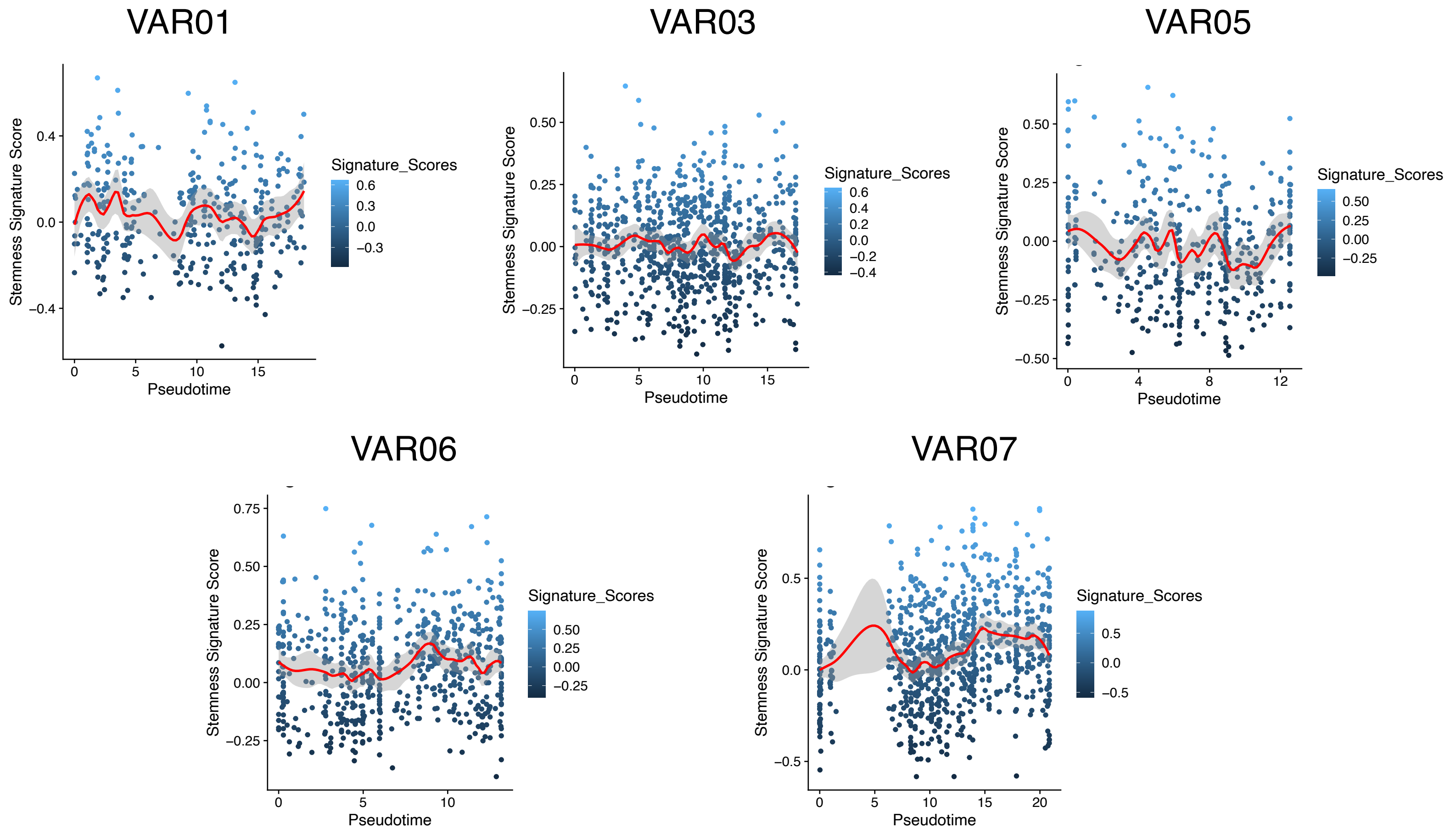

Supplementary Figure 9. Drug sensitivity heatmap of Top 100 most varied drugs within GDSC2 by tumor cluster.

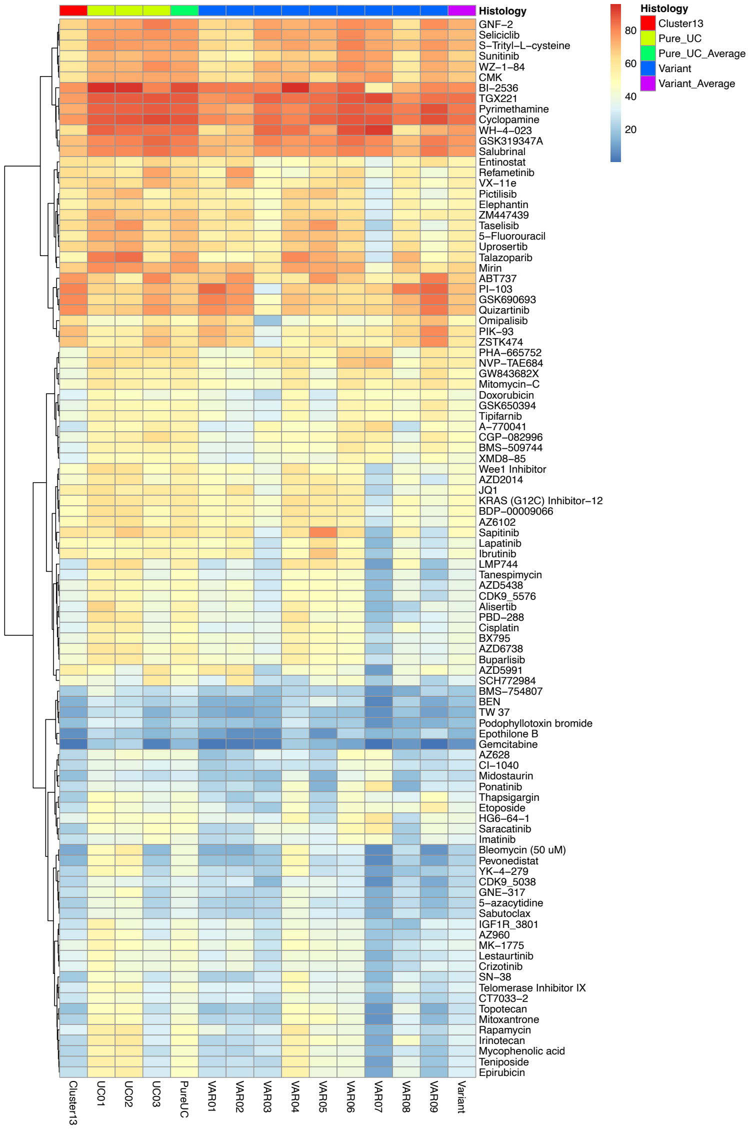

Supplementary Figure 10. Association of TM4SF1 with clinical features and luminal-basal subtypes in TCGA-BLCA. (A) Single set gene set enrichment analysis (ssGSEA) for TM4SF1 in TCGA-BLCA (N = 413 cases) . (B) TM4SF1 expression stratified by grade, stage, and lymph node status. (C) Kaplan-Meier curves showing overall survival according to TM4SF1 expression (pink = high, blue = low) in urothelial cancers, renal cancers, and pancreatic cancers.

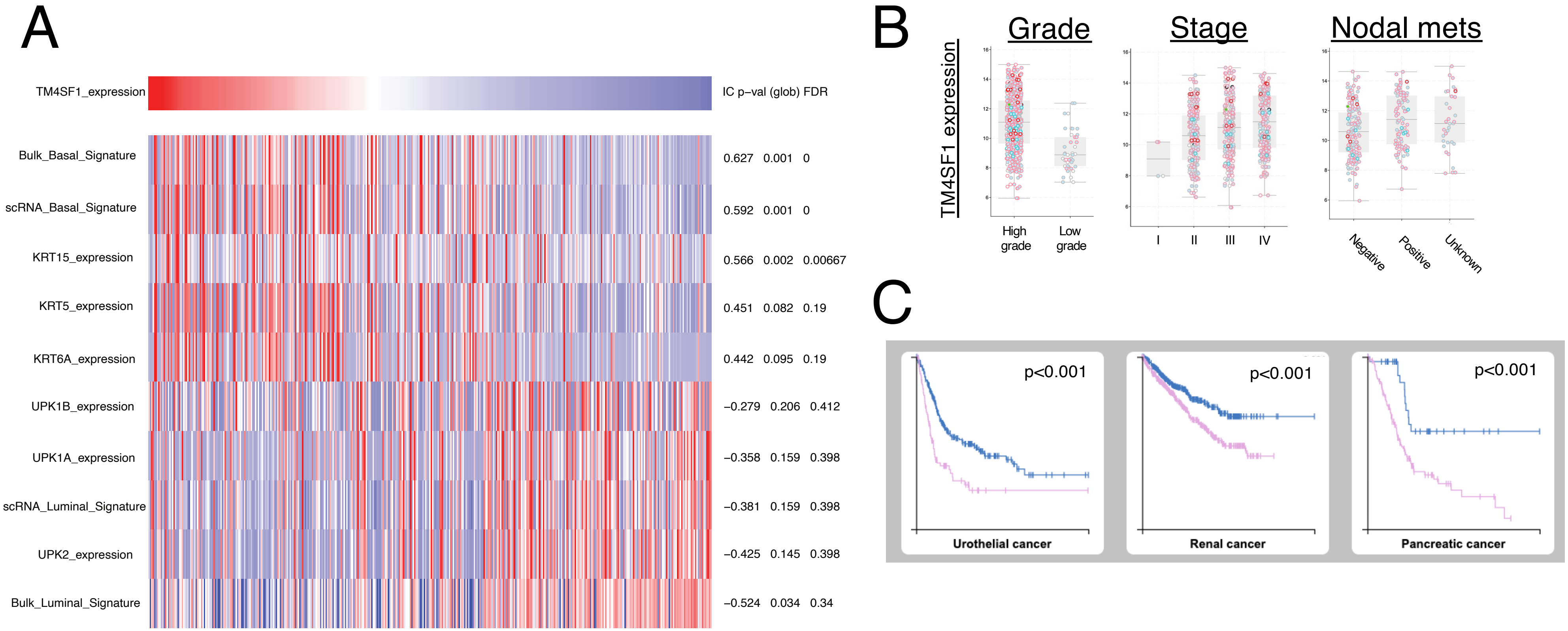

Supplementary Figure 11. Genes correlated with TM4SF1. (A) Scatter plot showing correlation coefficient and expression of genes associated with TM4SF1. (B-C) Correlation plots between TM4SF1 and CLDN4, EZR, EMP1, and KRT19 with linear regression within (B) tumor epithelial dataset and (C) by tumor. (D) ssGSEA in TCGA-BLCA (N = 413 cases) between TM4SF1 and EMP1, CLDN4, EZR, and KRT19.

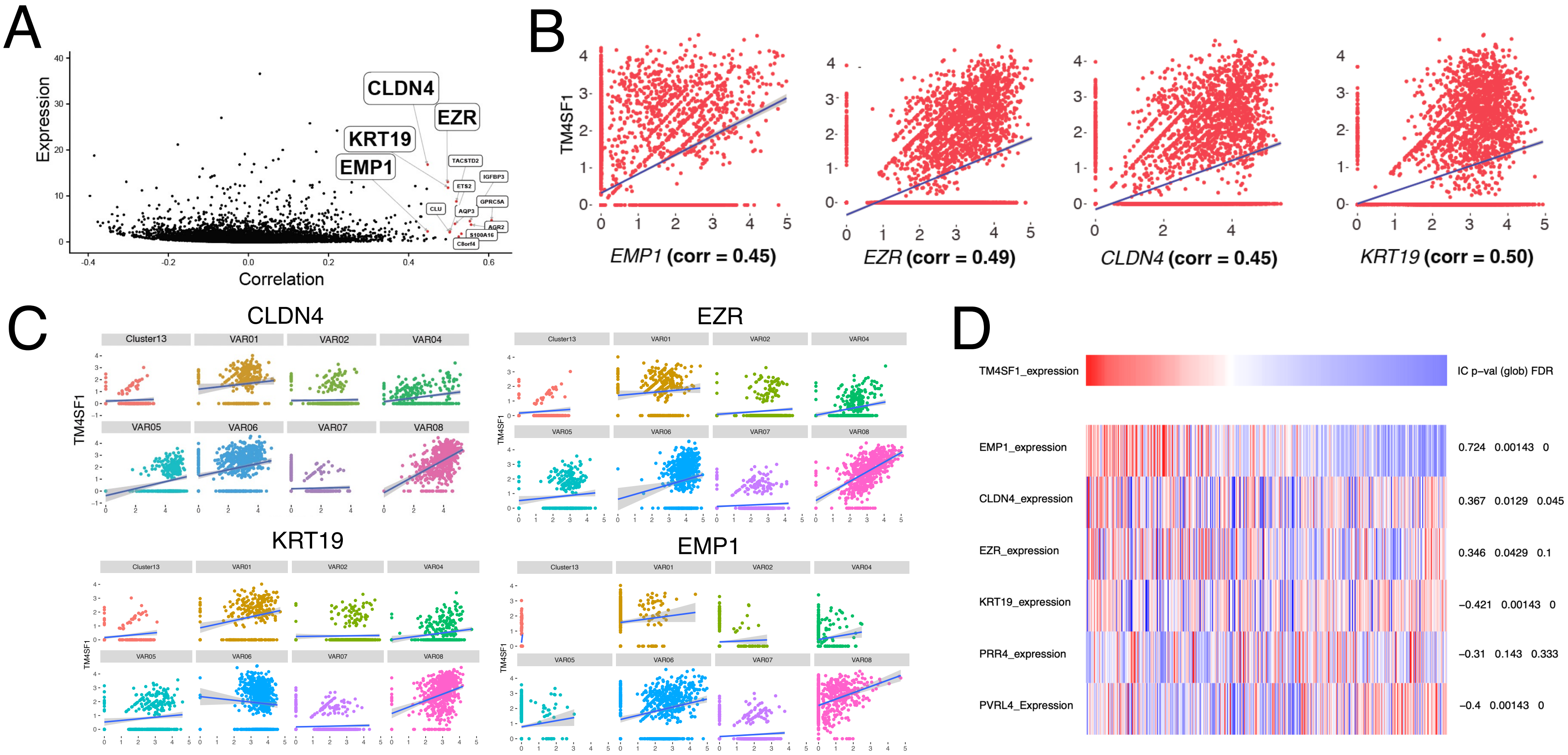

Supplementary Figure 12. Correlation between NECTIN4 and TM4SF1 in CCLE (*N* = 36 cell lines).

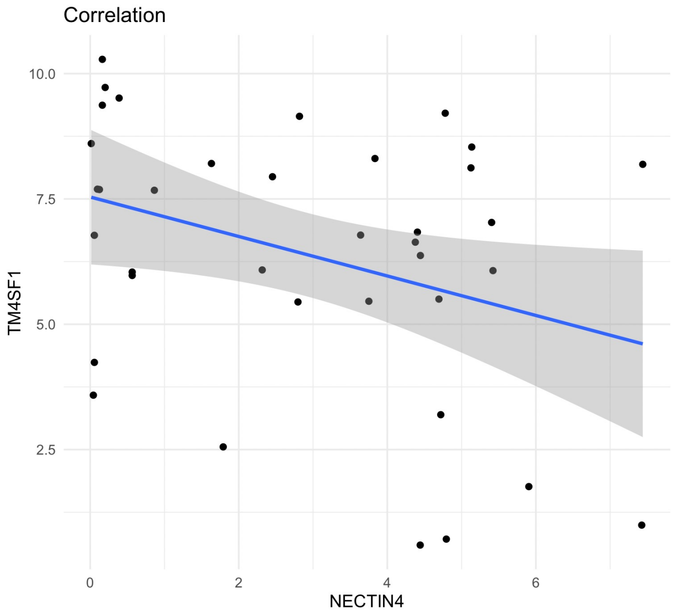

Cor = -0.35
p = 0.04

Supplementary Figure 13. Flow cytometric quantification CRISPR-Cas9-mediated TM4SF1 knockdown in UMUC3 cells. (A) Gating strategy to enrich for singlets. (B) Histogram of TM4SF1 surface expression in parental UMUC3 cells (cyan), UMUC3 TM4SF1-knock out (red), and negative control (gray). Abbreviations: SSC = side scatter, FSC = forward scatter.

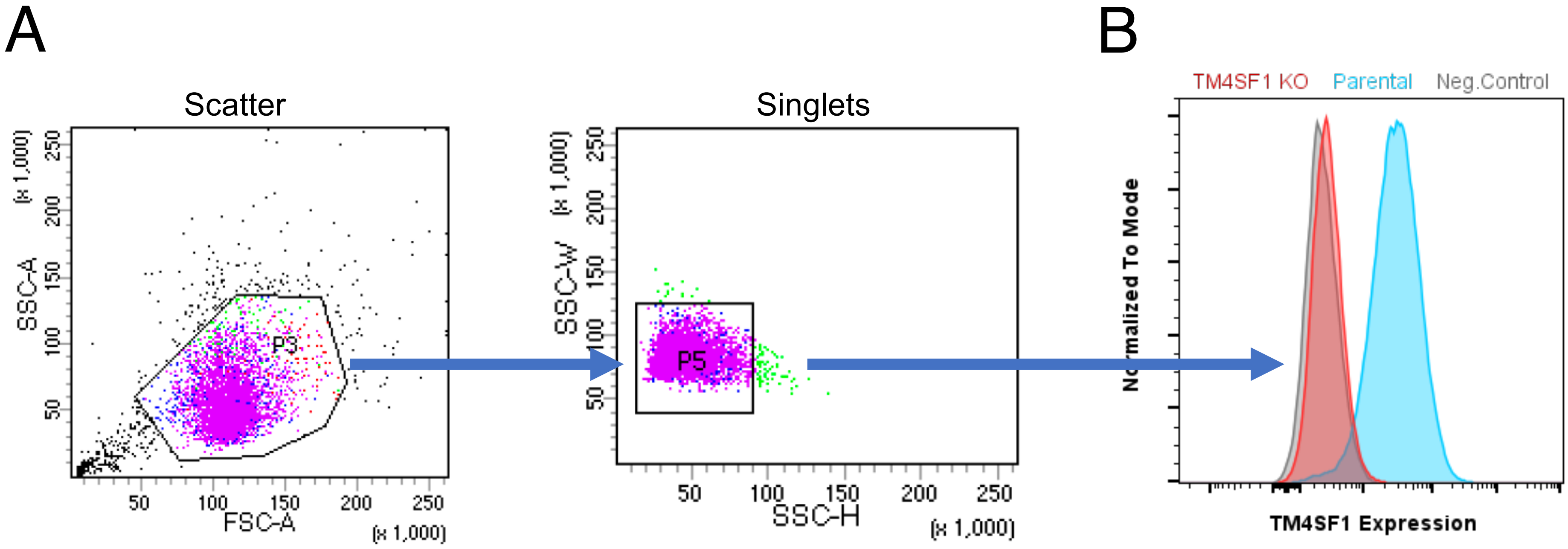

Supplementary Figure 14. Body weights of TM4SF1-CAR T treated UMUC3 mice.

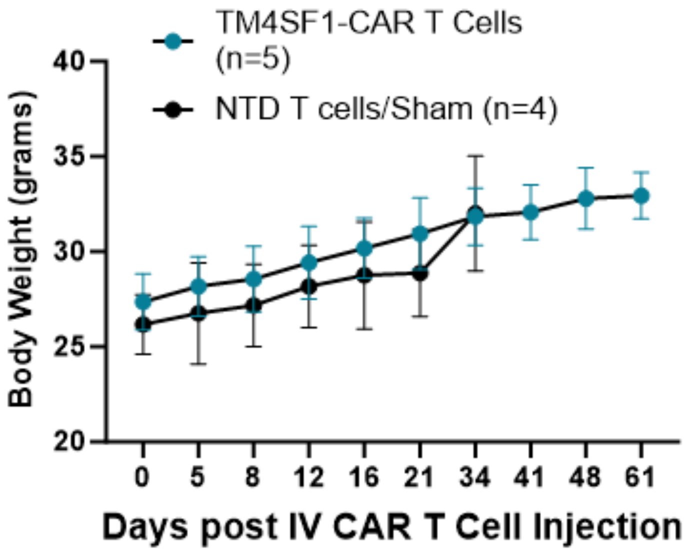

Supplementary Figure 15. TM4SF1 expression in normal tissues. Data obtained via GTExPortal. *TM4SF1*. Available from: https://www.gtexportal.org/home/gene/TM4SF1. Accessed April 3, 2025.

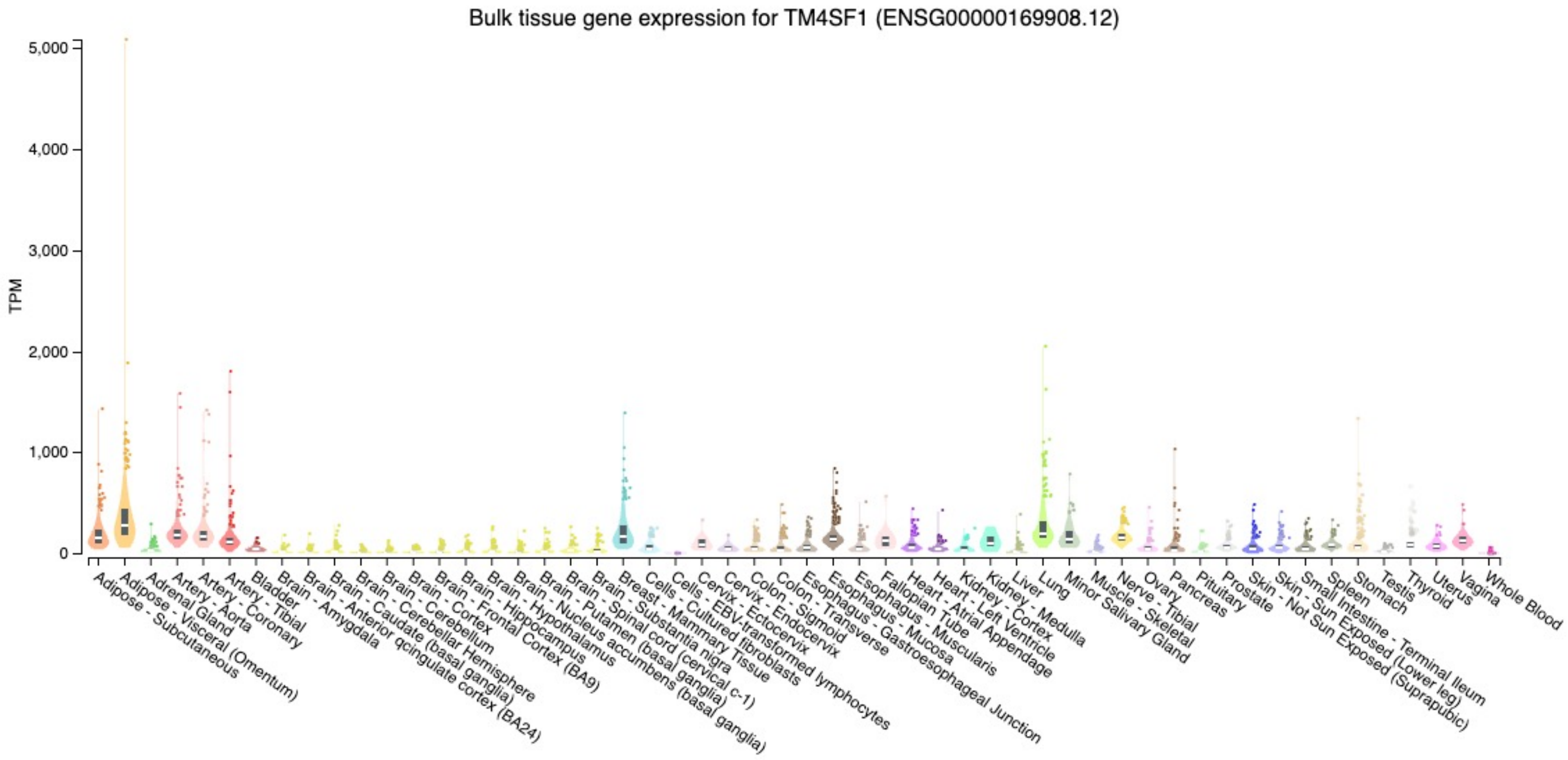

Supplement: Supplementary file 1 — Supplementary Information [file 41467_2025_59888_MOESM1_ESM.pdf]
