# Supplementary material for: Bladder cancer variants share aggressive features including a CA125+ cell state and targetable TM4SF1 expression
Source: Nat Commun. 2025 Jun 17;16:5312. doi: 10.1038/s41467-025-59888-8 (PMC12174346; doi:10.1038/s41467-025-59888-8)
Supplement: Supplementary file 2 — Reporting Summary [file 41467_2025_59888_MOESM2_ESM.pdf]

nature portfolio

Corresponding author(s): Franklin.W.Huang

Last updated by author(s): Apr 3, 2025

Reporting Summary

Nature Portfolio wishes to improve the reproducibility of the work that we publish. This form provides structure for consistency and transparency in reporting. For further information on Nature Portfolio policies, see our Editorial Policies and the Editorial Policy Checklist.

Statistics

For all statistical analyses, confirm that the following items are present in the figure legend, table legend, main text, or Methods section.

n/a	Confirmed	
☐	☒	The exact sample size (*n*) for each experimental group/condition, given as a discrete number and unit of measurement
☐	☒	A statement on whether measurements were taken from distinct samples or whether the same sample was measured repeatedly
☐	☒	The statistical test(s) used AND whether they are one- or two-sided<br>*Only common tests should be described solely by name; describe more complex techniques in the Methods section.*
☐	☒	A description of all covariates tested
☐	☒	A description of any assumptions or corrections, such as tests of normality and adjustment for multiple comparisons
☐	☒	A full description of the statistical parameters including central tendency (e.g. means) or other basic estimates (e.g. regression coefficient) AND variation (e.g. standard deviation) or associated estimates of uncertainty (e.g. confidence intervals)
☐	☒	For null hypothesis testing, the test statistic (e.g. *F*, *t*, *r*) with confidence intervals, effect sizes, degrees of freedom and *P* value noted<br>*Give P values as exact values whenever suitable.*
☒	☐	For Bayesian analysis, information on the choice of priors and Markov chain Monte Carlo settings
☒	☐	For hierarchical and complex designs, identification of the appropriate level for tests and full reporting of outcomes
☒	☐	Estimates of effect sizes (e.g. Cohen's *d*, Pearson's *r*), indicating how they were calculated

Our web collection on statistics for biologists contains articles on many of the points above.

Software and code

Policy information about availability of computer code

Data collection

Sequencing results were returned as paired FASTQ reads and processed with FastQC (v0.11.9) for general quality checks in order to further improve our experimental protocol. Then, the paired FASTQ files were aligned against the reference genome using a STAR aligner in the dropseq workflow (https://cumulus.readthedocs.io/en/latest/drop_seq.html). The aligning pipeline output included aligned and corrected bam files, two digital gene expression (DGE) matrix text files (a raw read count matrix and a UMI-collapsed read count matrix where multiple reads that matched the same UMI would be collapsed into one single UMI count) and text-file reports of basic sample qualities such as the number of beads used in the sequencing run, total number of reads, alignment logs.

Data analysis

Sequencing and alignment
Sequencing results were returned as paired FASTQ reads. These paired FASTQ files were then aligned against the hg19 reference genome (GRCh37.p13) using the dropseq workflow (https://cumulus.readthedocs.io/en/latest/drop_seq.html). The alignment pipeline output for each pair of FASTQ files included an aligned and corrected bam files, a digital gene expression (DGE) matrix text which was used for downstream analysis, and text-file reports of basic sample qualities such as the number of beads used in the sequencing run, total number of reads, alignment logs.

Single-cell quality control and clustering analysis
Cells were clustered and analyzed using Seurat (v4.3.0) in R (v.4.3.1). Cells with fewer than 300 genes, 500 transcripts, or a mitochondrial gene content of 20% or greater were removed. Doublets were removed using DoubletFinder (v.2.0.3). UMI-collapsed read-count matrices for each cell were used for clustering analysis in Seurat. We followed a standard workflow by using the “LogNormalize” method that normalized the gene expression for each cell by the total expression, multiplying by a scale factor 10,000. To identify different cell types, we computed the standard deviation for each gene and returned the top 2,000 most variably expressed genes among the cells before applying a linear scaling by shifting the expression of each gene in the dataset so that the mean expression across cells was 0 and the variance was 1. Principal

components analysis (PCA) was run using the previously determined most variably expressed genes for linear dimensional reduction and the first 100 principal components (PCs) were stored, which accounted for 47.04% of the total variance. For graph-based clustering, the top 75 PCs and a resolution of 0.5 were selected, yielding 36 cell clusters. Differentially expressed genes (DEGs) in each cluster were identified using the FindAllMarker function within the Seurat package and a corresponding p-value was given by the Wilcoxon's Rank Sum test followed by an FDR correction. In the downstream analysis, tumor cells from each patient were further clustered in a similar manner. For the individual patient clustering analysis, the number of PCs was determined by the statistical permutation test and the straw plot, and clustering resolution was selected accordingly.

Cell-type annotation and copy number variation
To annotate each cell type from the previous clustering, we referred to canonical markers and signature gene sets developed from established studies for each cell type. We computed the signature scores of these established gene sets for each cell in our dataset using the AddModuleScore function in Seurat. Each cluster in our dataset was assigned with an annotation of its cell type by top signature scores within the cluster. To validate the identities of the tumor cell populations, we estimated copy number variants (CNV) via InferCNV (Version 1.4.0), using all the non-tumor populations as reference. During the inferCNV run, genes expressed in fewer than five cells were filtered from the data set and the cut off was fixed at 0.1. Hidden Markov model (HMM) based CNV prediction was generated and estimated CNV events were shown in a heatmap.

Pseudotime analysis
To further investigate the differential trajectories of tumor cells in each patient, we conducted a pseudotime analysis in Monocle3. To analyze gene expression relative to the Cluster 13 cell state, Cluster 13 cells were selected as the starting point for the pseudotime trajectory. Pseudotime trajectories were computed accordingly and visualizations were made to illustrate specific gene expression levels along the pseudotime trajectory in each patient.

Gene ontology and gene set enrichment analysis
Within the tumor cells, we created a customized gene set signature for each variant tumor cell population of interest. Using the DEGs obtained from FindAllMarker function, we included genes with log2 fold change > 2 and statistical significance (FDR q < 0.05) in the customized signature gene set.

To assess the in silico functional roles of Cluster 13 cells, we used the signature gene sets derived from the scRNA-seq data to run gene ontology (GO) analysis against known signature gene set collections such as Hallmark, C2CP, C2CGP, C5GO and C6 oncogene collections (https://www.gsea-msigdb.org/gsea/msigdb/). The gene ratio and statistical significance levels from the overexpression test were calculated. Normalized gene expression data and variant tumor types as metadata were used in the GSEA analysis run on the GSEA software.20,21

To examine the association between signature gene sets or marker expression derived from our dataset and known basal/luminal signatures or canonical marker expression in the TCGA-BLCA bulk RNA sequencing dataset for validation, we performed ssGSEA (single set Gene Set Enrichment Analysis) by projecting the TCGA sample expression data onto the transcriptomic space defined by marker expression and established signature gene sets. For each target marker expression of target signature gene set, association was quantified via IC (information coefficient) and statistical significance was computed.

For manuscripts utilizing custom algorithms or software that are central to the research but not yet described in published literature, software must be made available to editors and reviewers. We strongly encourage code deposition in a community repository (e.g. GitHub). See the Nature Portfolio guidelines for submitting code & software for further information.

Data

Policy information about availability of data

All manuscripts must include a data availability statement. This statement should provide the following information, where applicable:

- Accession codes, unique identifiers, or web links for publicly available datasets
- A description of any restrictions on data availability
- For clinical datasets or third party data, please ensure that the statement adheres to our policy

All relevant datasets in this study are reported in the data availability section of the manuscript.

Research involving human participants, their data, or biological material

Policy information about studies with human participants or human data. See also policy information about sex, gender (identity/presentation), and sexual orientation and race, ethnicity and racism.

Reporting on sex and gender	All self-reported patient demographics (age, sex, race) were collected at time of surgery and enrollment into our study. Gender data were not available. Due to the low overall sample size, sex- and gender-based analyses were not deemed relevant to our study design. Consent was obtained for sharing of individual-level data for the 15 patients whose tumors were sequenced for this study.
Reporting on race, ethnicity, or other socially relevant groupings	Self-reported race was included in the demographic information for each patient. These data were included to provide representative information for the patient population at our institution.
Population characteristics	The participants in our study are representative of the patient population diagnosed with bladder cancer at UCSF. Due to the small sample size, we cannot make any remarks about how demographic characteristics relate to our findings.
Recruitment	All patients were recruited during preoperative counseling for bladder cancer surgery. Given the high enrollment rate (>90%) under this IRB-approved tissue-banking study, we do not believe there was any significant potential for self-selection bias.

Ethics oversight	All human subjects were enrolled in an institutional IRB-approved protocol (10-04057).

Note that full information on the approval of the study protocol must also be provided in the manuscript.

Field-specific reporting

Please select the one below that is the best fit for your research. If you are not sure, read the appropriate sections before making your selection.

☒ Life sciences ☐ Behavioural & social sciences ☐ Ecological, evolutionary & environmental sciences

For a reference copy of the document with all sections, see nature.com/documents/nr-reporting-summary-flat.pdf

Life sciences study design

All studies must disclose on these points even when the disclosure is negative.

Sample size	We obtained a total of 15 fresh bladder tumor samples from patients undergoing surgery at our institution under IRB 10-04057.
Data exclusions	QC was conducted in our analytical pipeline to remove low-quality cells. Cells with less than 300 genes, 500 transcripts, or a mitochondrial level of 20% or greater
Replication	Processed single-cell RNA sequencing data needed for reproducibility of our analytical findings will be deposited in the NCBI GEO database to ensure replication of the analysis.
Randomization	Samples were not allocated into experimental groups.
Blinding	Blinding was not relevant to this study.

Reporting for specific materials, systems and methods

We require information from authors about some types of materials, experimental systems and methods used in many studies. Here, indicate whether each material, system or method listed is relevant to your study. If you are not sure if a list item applies to your research, read the appropriate section before selecting a response.

Materials & experimental systems

n/a	Involved in the study	
☐	☒	Antibodies
☒	☐	Eukaryotic cell lines
☒	☐	Palaeontology and archaeology
☒	☐	Animals and other organisms
☐	☒	Clinical data
☒	☐	Dual use research of concern
☒	☐	Plants

Methods

n/a	Involved in the study	
☒	☐	ChIP-seq
☒	☐	Flow cytometry
☒	☐	MRI-based neuroimaging

Antibodies

Antibodies used	Hematoxylin and eosin (H&E) staining was performed using a standard method. CA125 (Signet, clone OC125) immunohistochemistry (IHC) was performed on an automated Ventana Benchmark Ultra IHC system using CC1 cell conditioning solution. MUC4 (CellMarque, 406M-15) and KRT24 (ThermoFisher, MA5-26581) IHC were performed at 1:50 and 1:5000 dilution, respectively, with 30-min antigen retrieval in EDTA pH 9.0 at 100 °C; TM4SF1 IHC was performed using a rabbit polyclonal antibody (Abcam, ab113504) at a 1:500 dilution after a 10-minute citrate antigen retrieval at 100 °C on a Leica Bond III platform.
Validation	All antibodies used in this study were obtained from commercial source, and validated according to manufacturers' instruction.

Clinical data

Policy information about clinical studies

All manuscripts should comply with the ICMJE guidelines for publication of clinical research and a completed CONSORT checklist must be included with all submissions.

Clinical trial registration	n/a
Study protocol	n/a

Data collection	n/a
Outcomes	n/a

Plants

Seed stocks	n/a
Novel plant genotypes	n/a
Authentication	n/a
